# Epi-drugs in combination with immunotherapy: a new avenue to improve anticancer efficacy

**DOI:** 10.1186/s13148-017-0358-y

**Published:** 2017-05-30

**Authors:** Roberta Mazzone, Clemens Zwergel, Antonello Mai, Sergio Valente

**Affiliations:** 1 Dipartimento di Chimica e Tecnologie del Farmaco, Sapienza Università di Roma, P.le Aldo Moro 5, 00185 Rome, Italy; 2 Center for Life Nano Science@Sapienza, Italian Institute of Technology, Viale Regina Elena 291, 00161 Rome, Italy; 3 Istituto Pasteur-Fondazione Cenci Bolognetti, Sapienza Università di Roma, P.le Aldo Moro 5, 00185 Rome, Italy

**Keywords:** Cancer, Epigenetics, Immunotherapy, HDAC inhibitors, DNA methylation, Immune checkpoint inhibitors

## Abstract

Immune checkpoint factors, such as programmed cell death protein-1/2 (PD-1, PD-2) or cytotoxic T lymphocyte-associated antigen-4 (CTLA-4) receptors, are targets for monoclonal antibodies (MAbs) developed for cancer immunotherapy. Indeed, modulating immune inhibitory pathways has been considered an important breakthrough in cancer treatment. Although immune checkpoint blockade therapy used to treat malignant diseases has provided promising results, both solid and haematological malignancies develop mechanisms that enable themselves to evade the host immune system. To overcome some major limitations and ensure safety in patients, recent strategies have shown that combining epigenetic modulators, such as inhibitors of histone deacetylases (HDACi) or DNA methyltransferases (DNMTi), with immunotherapeutics can be useful. Preclinical data generated using mouse models strongly support the feasibility and effectiveness of the proposed approaches. Indeed, co-treatment with pan- or class I-selective HDACi or DNMTi improved beneficial outcomes in both in vitro and in vivo studies. Based on the evidence of a pivotal role for HDACi and DNMTi in modulating various components belonging to the immune system, recent clinical trials have shown that both HDACi and DNMTi strongly augmented response to anti-PD-1 immunotherapy in different tumour types. This review describes the current strategies to increase immunotherapy responses, the effects of HDACi and DNMTi on immune modulation, and the advantages of combinatorial therapy over single-drug treatment.

## Background

Accumulation of genetic alterations might be caused by abnormal expression of genes that play a key role in regulation of cell survival, proliferation, and death. In addition, many studies have evaluated the capability of epigenetic regulators to modulate gene expression in cancer cells through covalent modification of DNA as well as histone and non-histone proteins [1]. The most important epigenetic processes reported in the clinical settings involve DNA methylation and histone modifications without altering the DNA sequence of bases. DNA methyltransferases (DNMTs) catalyse the methylation at cytosine-C5 mainly in a CpG dinucleotide context at the promoters of selected genes [2]. Although DNA methylation is essential for fundamental processes like embryonic development or differentiation, aberrant expression and/or activities of DNMTs are involved in several pathologies, from neurodegeneration to cancer [3–6]. DNMT enzymes are classified into three distinct families: DNMT1; DNMT2, also known as TRDMT1 (t-RNA cytosine-5-methyltransferase); and DNMT3 (consisting of DNMT3a, DNMT3b, and DNMT3L) [2, 7]. Currently, two DNMT inhibitors (DNMTi), the nucleoside analogues azacitydine (5-AZA) and decitabine (5-AZA-CdR), have been approved by FDA and the European Medicines Agency (EMA) against myelodysplastic syndromes (MDS), acute myeloid leukaemia (AML), and chronic myelomonocytic leukaemia (CMML). DNMT overexpression is described in numerous cancer types. DNMTi can arrest tumour growth and cell invasiveness and can induce cell differentiation [8]. Among histone-modifying enzymes, histone acetyltransferases (HATs) and histone deacetylases (HDACs) are among the most studied targets for chromatin remodelling, control of gene expression, and anticancer therapy. HDACs are divided into four groups: “classical HDACs” are expressed in the nucleus and/or cytoplasm, share a Zn^2+^-dependent catalytic activity, and include class I (HDAC1, 2, 3, 8), IIa (HDAC4, 5, 7, 9), IIb (HDAC6 and 10), and IV (HDAC11) enzymes. Class III HDACs, known as sirtuins, possess NAD^+^-dependent deacetylase activity and share no sequence similarity with the classical deacetylases [9]. HDAC inhibitors (HDACi) can induce, among others, tumour cell apoptosis, growth arrest, differentiation, inhibition of angiogenesis, and immunogenicity [10]. Among them, vorinostat and romidepsin have been approved for treatment of refractory cutaneous T cell lymphoma (CTCL), belinostat and chidamide (the latter approved only in China) for peripheral T cell lymphoma (PTCL), and panobinostat for multiple myeloma (MM), all from 2006 to 2015. Interestingly, most HDACi and DNMTi have shown a potent immunomodulatory activity, thus justifying their application in cancer immunotherapies. In fact, there is a growing interest in understanding how these potential therapies can modulate the host immune system in order to achieve beneficial antitumour effects [11]. The cancer immunotherapy field is under intense investigation to ameliorate cancer cell recognition by immune cells and to make them more sensitive to cytotoxic antitumour treatment. Cancer immunotherapy refers to a diverse range of therapeutic approaches to improve the capability of T cells and other immune effector cells in recognition and elimination of cancer cells through overcoming of cancer cell resistance in different tumour types [12]. Moreover, cancer cell immune recognition provides the tools to modulate immune signalling pathways that drive tumour growth and progression, suggesting rational combinatorial approaches [13]. This review will also focus on current immunomodulatory combinatorial treatment strategies aiming to improve the effectiveness of cancer immunotherapy.

## Cancer cell escape mechanisms and the role of the immune system

Actually, cancer immunotherapy strategies designed to break the immune tolerance can be broadly classified on the basis of the mechanisms involved in resistance processes. Such strategies include several factors: (i) adoptive transfer of immune effectors, (ii) vaccination, and (iii) immunomodulatory therapy. In particular, effector cells of innate immunity, such as natural killer (NK) cells and macrophages, and adaptive immunity (such as T and B cells) can eliminate immunogenic malignant cells [14]. Nevertheless, the main escape pathways, including anti-apoptotic signalling, mitogen-activated protein kinase (MAPK) pathway, microphthalmia-associated transcription factor (MITF), cyclic adenosyl monophosphate (cAMP), and nuclear factor kappa light chain enhancer of activated B cells (NF-κB)-related mechanisms, negatively influence the therapeutic success. Part of this failure is due to immune suppression by the tumour microenvironment (TME). So far, defective antigen presentation, tumour-induced inhibitory checkpoint pathways against effector T cell activity, infiltrating regulatory T cells (Tregs), myeloid-derived suppressor cells (MDSCs), and secretion of immunosuppressive cytokines, such as transforming growth factor β (TGF-β), interleukin-6 (IL-6), and vascular endothelial growth factor (VEGF), represent the major mechanisms for escaping [15]. It is well known that the immune system protects the host against tumour development on one side and promotes tumour growth by selecting tumours of lower immunogenicity on the other side. These two effects create a dynamic process also called “cancer immunoediting” that includes three phases: elimination, equilibrium, and escape [16]. However, due to their heterogeneity, tumour cells with a less immunogenic phenotype are able to escape this elimination phase also called immunosurveillance and to expand during the equilibrium phase. These considerations have encouraged many researchers to develop new therapeutic strategies to fight different cancer types with immunotherapy.

## Rationale for the development of cancer immunotherapy strategies

Active research in tumour immunology includes studies on adoptive T cell therapy and cancer vaccination, as well as clinical investigation regarding immune checkpoint blockade in combination therapy.

The immune system plays a key role in maintaining self-tolerance and regulating T cell responses. For this reason, it is very important to understand the complex and dynamic nature of host immune responses and the regulation of additional molecules in the TME in order to develop strategies to improve clinical efficacy. Activation of antigen-specific T cells is a key step in immune responses, and it is provided by the interaction between the peptide-major histocompatibility complex (MHC) complex and the T cell receptor (TCR) in the presence of other co-stimulatory molecules. Among these molecules, cluster of differentiation 28 (CD28), expressed on the surface of naive CD4^+^ and CD8^+^ cells, is one of the most important proteins involved in the initial activation of the immune system response.

Conversely, the interaction between molecule and antigenic peptide in the absence of co-stimulation results in T cell “anergy” instead of activation [17]. Immune system homeostasis includes the presence of both stimulatory and inhibitory signals such as cytotoxic T lymphocyte-associated antigen-4 (CTLA-4, a CD28 homolog), which acts by a competitive mechanism with CD28 for binding to its cognate ligands such as CD80/86 expressed on the surface of antigen-presenting cells (APCs) [18]. Another important immune checkpoint is mediated by programmed cell death protein-1 (PD-1). In comparison with CTLA-4, PD-1 regulates immune activity when effector T cell tissue infiltration occurs. Besides activated T cells, PD-1 is mainly expressed on the surface of activated B cells, NK cells, dendritic cells (DCs), and Tregs [13]. Engagement of PD-1 by its ligands, either PD-L1 or PD-L2, induces a negative control signal resulting in the inhibition of T cell proliferation, cytokine production, and cytotoxic activity [19]. Moreover, upregulation of PD-L1 on different tumour types and production of cytokines as a consequence of inflammatory signals induces an innate (tumour cell intrinsic) and an adaptive resistance, respectively. Preclinical studies have demonstrated that blocking the PD-L1/PD-1 interaction augments antitumour T cell responses [20]. About 20% of advanced non-small cell lung cancer (NSCLC) patients and 30–40% of advanced melanoma patients have provided tumour responses to PD-1 blockade monotherapy [21]. Therefore, modulation of immune inhibitory pathways is considered as an important breakthrough in cancer treatment. In particular, since 2011 with the approval by FDA of the monoclonal antibody (MAb) ipilimumab for advanced melanoma, and 3 years later of pembrolizumab and nivolumab as well, there has been an increasing interest in this field. Notably, ipilimumab, targeting CTLA-4 on T cells, allows T cell activation for immune responses in several cancers as well as inhibition of Treg function [22]. Early clinical trials evaluated ipilimumab in patients with a variety of malignancies, including melanoma, prostate cancer, renal cell carcinoma, and non-Hodgkin lymphoma [23, 24]. Similarly, an intense investigation has been conducted for nivolumab and pembrolizumab, fully human and humanized IgG4 anti-PD-1 MAbs, respectively [25, 26]. In general, different tumour type responses to checkpoint blockade are more closely associated with inherent immunogenicity (mutational burden or dominant neoantigens) than with the tumour tissue origin [27]. In preclinical models, combined blockade of PD-1 and CTLA-4 achieved more pronounced antitumour activity than blockade of either pathway alone [28–31]. Indeed, the first reported ipilimumab and nivolumab combination with response in melanoma has provided a rationale for the development of immune checkpoint combination strategies (NCT01024231) [32]. Additionally, recent studies have shown a synergistic antitumour activity in mouse MC38 and CT26 colorectal tumour models with concurrent, but not sequential, CTLA-4 and PD-1 blockade (ipilimumab and nivolumab) [33]. Updated reviews about the current status of immunotherapy and clinical developments of immune checkpoint inhibitors have been recently reported [34–36].

## Epigenetic regulation of the immune system

Immune checkpoint regulation mechanisms include covalent modifications, microRNAs (miRNAs), long noncoding RNAs (lncRNAs), and histone modifications [37]. Epigenetic modifiers can either turn on or turn off immune responses, resulting in immune evasion [38]. Since some epigenetic regulators have shown a potent immunomodulatory activity, their combination with immune checkpoint inhibitors could represent a promising therapeutic strategy. Currently, many researchers are investigating the link between epigenetic modulation of the immune system and cancer development. Among the epigenetic processes implicated in immune regulation, DNA methylation and histone acetylation are likely the most important modifications in controlling development, differentiation, and functions of T cells [39]. During immune responses, naive CD4^+^ T cells differentiate into several T helper (Th) cell subsets, including Th1, Th2, Th17, and induced regulatory T (iTreg) cells, as defined by their pattern of cytokine production [40]. Moreover, CD4^+^ Th subsets are distinguished by their phenotype as well as by the transcription factors that control their differentiation, including T-bet in Th1, GATA-3 in Th2, RAR-related orphan receptor γ (RORγT) in Th17, and forkhead box protein 3 (Foxp3) in Treg [41]. The first studies in humans showed that Th1 and Th2 cells are true lineages regulated by epigenetic modifications occurring on interferon-γ (*IFN-γ*), *IL-4*, and *IL-13* genes. The *IFN-γ* promoter is hypermethylated in human naive T cells and is demethylated during the differentiation to Th1 cells [42]. Conversely, Th2 cell differentiation results in the selective demethylation of several specific CpG dinucleotides in the *IL-4* and *IL-13* genes, which are expressed in activated Th2 but not Th1 cells [43]. Moreover, epigenetic histone marks are also essential for the Th1/Th2 cell fate decisions. Signal transducer and activator of transcription 4 (STAT4) and T-bet or STAT6 and GATA-3 are key transcription factors for the Th1 and Th2 lineages, respectively [44]. The histone methyltransferase (HMT) SUV39H1, which is involved in H3K9 trimethylation (H3K9me3), has recently been implicated in the silencing of the Th1 locus and the subsequent promotion of stability of Th2 cells [45]. Chang et al. explored the mechanisms establishing long-range H4 acetylation marks at the *IFN-γ* locus, during Th1 lineage commitment. T-bet displaced the Sin3 transcription regulator family member A (Sin3A)-histone deacetylase (HDAC1, HDAC2) complexes, to facilitate the differentiation of Th1 cells [46]. In response to IL-12 signals, the activation of STAT4 required for the development of Th1 cells facilitates chromatin remodelling at the enhancer regions of *Th1* genes. Similarly, Th2 commitment requires STAT6 and GATA-3 activities in response to IL-4 stimulation [47]. Therefore, transcription factors not only promote T cell differentiation but also influence epigenetic states and gene expression programs that define a particular lineage. Furthermore, epigenetic histone modifications by enhancer of zeste homolog 2 (EZH2), a member of polycomb repressive complex 2 (PRC2), regulate differentiation and plasticity of CD4^+^ T cells. Notably, EZH2 directly binds and facilitates correct expression of T-box transcription factor 21 (Tbx21) and GATA-3 for differentiating Th1 and Th2 cells, accompanied by increased H3K27 trimethylation (H3K27me3) [48]. Finally, in Tregs, Foxp3 is acting predominantly as a transcriptional repressor and is required for establishment of the chromatin repressive mark H3K27me3 in activated Tregs. Indeed, Foxp3 has been found to interact with EZH2 exclusively in activated Tregs, suggesting that Foxp3 recruits the PRC2 complex to target genes and forms repressive chromatin under inflammatory conditions [49]. Morinobu et al. analysed the histone acetylation levels of *Th1* genes, *IFN-γ*, *T-bet*, and *IL18RAP* in response to different cytokines [50]. Multiple levels of regulation of *IFN-γ* histone acetylation may reflect critical checkpoints for Th1 differentiation. In addition, basic leucine zipper transcription factor (BATF) regulates *Th1* gene expression via acetylation of *T-bet* and *IFN-γ*, considered as an important checkpoint in T cell differentiation [51]. Several other findings suggest that miRNA epigenetic modifications in cancer can promote an immune evasion [52]. More recently, Cortez et al. have identified a novel mechanism of PD-L1 epigenetic regulation by which tumour immune evasion is regulated by the p53/miR-34/PD-L1 axis [53]. Indeed, p53 influences immune response by monitoring T cell activation and inflammatory cytokines and enhancing tumour cell recognition by NK cells [54, 55]. Furthermore, the overexpression of T cell immunoglobulin and mucin domain 3 (Tim-3) on T cells negatively controls the antitumour T cell responses, with important implications for anti-PD1 immunotherapy [56]. Another important immune checkpoint is lymphocyte-activation gene 3 (LAG-3), highly expressed on activated T cells in many cancer types, that can be used as an immunotherapy target [57]. miR-138 has been reported with a multifaceted role in carcinomas, although its ability to interact with the immune system is unknown. Wei et al. have demonstrated that the combination of miR-138 with a MAb therapy against CTLA-4 provided a strong therapeutic synergism. Transfection of human CD4^+^ T cells with miR-138 suppressed expression of CTLA-4, PD-1, and Foxp3 in glioma preclinical models [58]. Moreover, previous studies described a novel biological role of other miRNAs in regulating the expression of immune checkpoints [59, 60]. Hence, targeting these miRNAs in combination with traditional immune checkpoint inhibitors is certainly a potent immunotherapeutic strategy. At last, lncRNAs are also critical mediators in various tumours associated with cancer progression [61, 62]. Notably, Zeng et al. have found that the nuclear paraspeckle assembly transcript 1 (NEAT1) expression was repressed by PML-RARα, a leukemic-specific antigen and part of the PD-1 pathway. Moreover, reduced NEAT1 expression may play a role in the myeloid differentiation of acute promyelocytic leukaemia (APL) cells [63]. Many lncRNAs are bound and regulated by the key T cell transcription factors T-bet, GATA-3, STAT4, and STAT6. Hu et al. have found that LincR-Ccr2-5′AS, together with GATA-3, is essential for regulation of several chemokine receptor genes and for Th2 cell migration, but the exact mechanism of action of LincR-Ccr2-5′AS is currently unknown [64].

## The potential role of epi-drugs as “immune-regulators”

HDACi are being used as a novel, therapeutic approach for treatment of leukaemia and other haematological malignancies [2, 65]. However, their effect on immune cells remains ill-defined, as HDACi may impair immune surveillance. Cancer arises as a result of accumulation of genetic mutations and epigenetic aberrations regulated by many players including HDACs. Abnormal expression of HDACs has been reported in tumours, whereas knockdown of HDACs inhibits tumour growth [66]. Tumour cell-intrinsic responses to HDACi treatment involving cell death, arrest of proliferation, and modulation of tumour immunogenicity have already been well described and reviewed [2, 67]. In particular, cell death is one of the deepest studied antitumour activity of HDACi, which are able to induce apoptosis by various pathways and processes, including activation of both intrinsic and extrinsic apoptosis pathways by modulating expression of pro- and anti-apoptotic genes, and by activating and/or inducing transcription factors such as E2F1, forkhead box protein O1 (FOXO1), p53, and specificity protein 1 (Sp1) [68, 69]. Another important mechanism by which HDACi can induce tumour cell death is the generation of reactive oxygen species (ROS) that decrease the expression of free radical scavengers. It has been reported that vorinostat and entinostat treatment can induce selective accumulation of ROS and caspase activation only in transformed cells [70]. Hui et al. have demonstrated that synergistic killing of gastric carcinoma (GC) cells by bortezomib/romidepsin combination was dependent on ROS generation and caspase activation. Collectively, this combinatorial effect could also induce autophagy by the activation of MAPK family members (ERK1/2 and JNK) [71]. Furthermore, a synergistic antiproliferative effect has been observed by combination treatment with vorinostat and gefitinib or erlotinib, two epidermal growth factor receptor (EGFR) tyrosine kinase inhibitors (TKIs), through reduction of cell migration in NSCLC cells. However, the key finding of this study is that the upregulation of the major mitochondrial porin, the voltage-dependent anion-selective channel protein 1 (VDAC1), by vorinostat and TKIs could be involved in oxidative stress-dependent apoptosis. In addition, the usage of vorinostat alone or in combination modulated the c-Myc-NRF2-KEAP1 pathway, crucial for the redox stress response [72]. Further important biological responses to HDACi include cell cycle arrest at the G1/S and G2/M checkpoints, cellular senescence, and autophagy. A recent study has shown that activation of FOXO1 transcription factor by HDACi is an important mediator of autophagic response [73]. HDACi have been recently tested in combination with immunotherapeutic approaches. In addition to their direct antitumour effects, these agents could facilitate recognition and sensitivity to effector functions by cytotoxic T lymphocytes (CTLs) and NK cells, thereby sensitizing cancer cells to immunotherapy. Conversely, in cancer patients, immunological side effects of HDACi such as lymphopenia, leukopenia, neutropenia, and thrombocytopenia might be contradictory for their application in cancer immunotherapy. On the other hand, there is an increasing number of studies showing beneficial effects and immunomodulatory properties of these agents. To date, a number of studies referring to the ability of HDACi in upregulating MHC, co-stimulatory molecule expression, components involved in tumour necrosis factor (TNF) superfamily signalling have been performed [74]. Nevertheless, the molecular mechanisms underlying the involvement of HDACi-regulated genes in immune recognition are not fully understood. Trichostatin A (TSA), a pan-HDACi, in combination with valproic acid (VPA), a class I/IIa HDACi, has been reported to enhance cell surface expression of class I MHC and co-stimulatory molecules CD40 and CD86 in melanoma cells [75]. In the same way, sodium butyrate, a class I/IIa HDACi, and TSA activated expression of class I and II MHC and CD40 in multiple human neuroblastoma (NB) or mouse plasmacytoma J558 tumour cell lines [76]. Furthermore, romidepsin promotes tumour-specific T cell-mediated killing of B16/F10 murine melanoma cells and enhances the expression of class II MHC, CD40, and B7-1/2 [77]. Many studies reported that HDACi sensitize tumour cells to NK cell lysis by promoting expression of NK cell ligands [78–80]. Moreover, low cell cytotoxicity by reducing the NK cell activation receptors has been documented using therapeutic concentration of vorinostat and VPA. In a further study, Rossi et al. have demonstrated the reduction of NK cell production by IFN-γ after TSA, VPA, and sodium butyrate treatment [81]. HDACi are also important for macrophage differentiation, polarization, and innate defence function [82]. Multiple studies showed a suppressive role of HDAC inhibition during macrophage activation status. Roger et al. have described that the blockage of class I and II HDACs enhances the recruitment of the repressive complex Mi-2b to the promoters of *M1* activation state genes, such as II6 [83]. Cabanel et al. have highlighted the role of TSA as a macrophage differentiation and elongation regulator. They assessed, for the first time, that macrophage plasticity is kept by HDAC inhibition. Furthermore, simultaneous inhibition of class I and II HDACs in several macrophage populations results in reduced levels of recognition receptors, activation markers, cytokines, and chemokines [84]. Moreover, HDAC inhibition can functionally target Tregs and helps to break the immune tolerance. Low levels of Tregs exist under normal physiological conditions, where they mediate the suppression of sustained inflammation, prevent autoimmune responses, and keep homeostasis of immune response. In cancer patients, Tregs are induced by tumour or stroma-secreted factors and also regulated by effector B, T cells, and OX40/OX40L expressed on activated CD4^+^ and CD8^+^ T cells, members of the TNFR/TNF superfamily [85, 86]. Tregs are capable of inhibiting NK and T cell function in TME, thus impairing both innate and tumour antigen-specific antitumour immune responses. Nowadays, it is well established that Foxp3 is the major key regulator of Treg development and function. Among the epigenetic modifications, acetylation, together with methylation, regulates the stability and activity of Foxp3 [87]. Furthermore, recent reports have described opposite mechanisms by which different HDAC isoforms modulate Treg and Treg-Foxp3 expression. For instance, by enhancing Foxp3 acetylation, entinostat has been found to increase Treg suppression function. The mechanism of Foxp3 expression regulation by entinostat may involve acetylation of STAT3 protein, which is a substrate of HDAC3 [88]. Conversely, other authors have shown Treg and Foxp3 downregulation following entinostat treatment [89]. Beier et al. suggested that Sirt1, HDAC6, or HDAC9 have different effects on Treg biology. Although HDAC inhibition increased the expression of the *Foxp3*-encoding gene, the transcription factors involved are different. In particular, loss of HDAC9 stabilizes STAT5 acetylation (K694, K701, and K359) and phosphorylation (Y694) and increases Treg function [90]. On the contrary, HDAC5 decreased Treg suppressive function and impairs iTreg formation as well as IFN-γ production [91]. Other researchers have investigated the effect of HDACi on suppressive myeloid cells, including myeloid-derived suppressor cells (MDSCs) that are comprised of monocytic (M-MDSC) and polymorphonuclear (PMN-MDSC) cells. Suppressive myeloid cells, also including tumour-associated macrophages (TAMs), are induced by tumour growth and accumulated in TME. These cells impair host immunity against tumour cells and facilitate tumour progression and metastasis. Youn et al. have reported that HDAC2 inhibitors can directly interact with the retinoblastoma 1 (Rb1) promoter and participate in silencing Rb1 expression in tumour-bearing hosts. This evidence suggested that HDAC2 inhibitors may regulate pathologic differentiation of myeloid cells in cancer [92]. It has been shown that treatment with TSA and vorinostat led to the expansion of MDSCs in bone marrow cells in vitro, and this effect has been confirmed also in vivo by TSA treatment [93]. Recently, to deeply explore the HDACi impact on antigen presentation, Tiper and Webb have provided evidence on combination of HDACi and NK T cell-based immunotherapy. Importantly, HDACi treatment not only enhances both CD1d- and class II MHC-mediated antigen presentation but also inhibits inflammatory cytokine secretion, which may contribute to the suppression of antitumour NK T cell responses. Moreover, the same authors demonstrated the efficacy of HDACi in restoring antitumour responses to mantle cell lymphoma (MCL) through both cell-intrinsic and cell-extrinsic mechanisms [94].

**Fig. 1:**
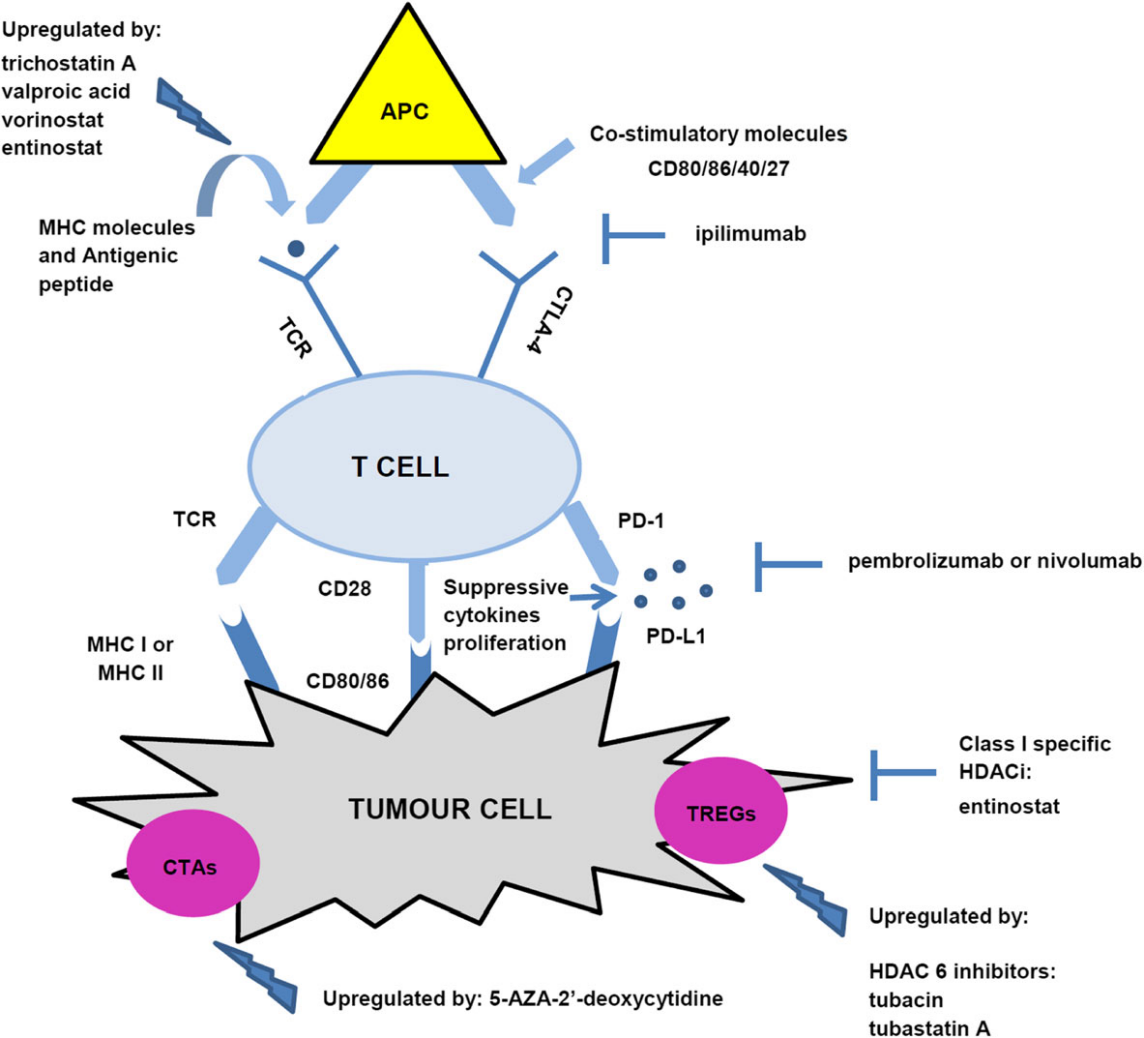
Interaction between tumour and immune cells. T cell stimulation is driven by antigens and requires a coordinated participation of several other receptors and molecules expressed on the T cell surface and antigen-presenting cells (APCs) or tumour cells. HDACi and/or DNMTi can inhibit different signalling pathways involved in adaptive immune responses, enhancing antitumour effects by combination with immune checkpoint inhibitors

Also, DNMTi seem to be implicated in the modulation of various immune system components, including tumour-associated antigen (TAA) and antigen presentation machinery (APM). Cancer testis antigens (CTAs) are a large family of tumour-associated antigens expressed in histologically different human tumours, but not in normal tissues except for the testis and placenta. CTAs include the melanoma-associated antigen (*MAGE*), *NY-ESO-1*, and *SSX* gene families and the GAGE/PAGE/XAGE superfamilies, and they are expressed by neoplastic cells and recognized by CTLs. These tumour-restricted expression patterns, together with their strong in vivo immunogenicity, identified CTAs as ideal targets for tumour-specific immunotherapeutic approach, and several clinical trials for a CTA-based vaccine therapy have been developed after these findings [95–97]. DNA methylation can lead to induction or upregulation of CTA expression in histologically different solid tumour cells, as well as in stem cells [98]. CTAs, such as preferentially expressed antigen in melanoma (PRAME), have been induced by pharmacological inhibition (5-AZA-CdR) or genetic knockdown of DNMTs, in epithelial ovarian cancer (EOC) or in HGSC [99]. DNMTi are capable of regulating APM on tumour cells through different mechanisms. APM plays an important role during the recognition phase and lysis of neoplastic cells by antigen-specific CTLs and represents a good candidate for immunotherapy likewise CTAs. In addition, to boost immune response, DNMTi can decrease immunosuppression by reducing Treg function [100]. Interestingly, DNMTi and HDACi were strongly effective in inducing upregulation of APM component expression in a broad spectrum of tumour types, suggesting a contribution by indirect epigenetic mechanisms not yet identified [101]. Recently, the role of histone methyltransferases (HMTs) in tumour immunity has been investigated. For instance, combination of EZH2 inhibitors such as deazaneplanocin A (DZNep) or tazemetostat (EPZ6438) with 5-AZA displayed improved therapeutic efficacy of anti-PD-L1 treatment by increasing Teff tumour infiltration and decreasing tumour progression [102]. EZH2 and other PRC2 components have been found to repress the expression of CXCL9 and CXCL10 (Th1-type chemokines) even in colon cancer [103]. However, initial support for the immunological efficacy of DNMTi, alone or combined with HDACi, came out from studies in haematological malignancies, such as AML and MDS, for which 5-AZA and 5-AZA-CdR have been approved by FDA, as already mentioned. In the last decade, a clinical study has reported that the administration of 5-AZA-CdR with VPA induces anti-MAGE CD8^+^ response in 50% of patients with AML (Fig. 1) [104].

**Fig. 2:**
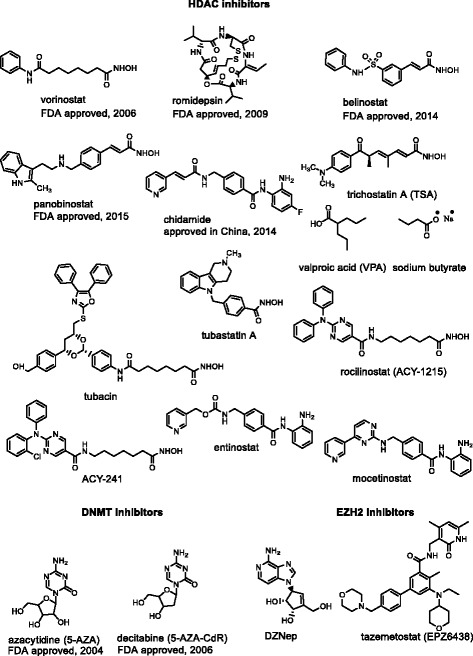
Structures of epi-drugs discussed in this review

The structures of the epi-drugs discussed in the above section are shown in Fig. 2.

## Preclinical studies of HDACi and DNMTi in combination with immunotherapies

In this section, main preclinical studies are described involving immunotherapy strategies in combination with HDACi or DNMTi (Fig. 2). As aforementioned, HDACi can enhance T cell survival and function and induce expression of multiple chemokines in tumour cells, tumour-infiltrating macrophages, and T cells, thus augmenting the response to anti-PD-1 immunotherapy in lung adenocarcinoma [105]. Over the last decade, extensive studies have been carried out to evaluate the efficacy of combining HDACi with various immunotherapy treatments to overcome cancer cell resistance and strongly improve clinical responses.

Recently, Kim et al. have reported that treatment with both anti-PD-1 and anti-CTLA-4 antibodies was unable to eradicate two types of immunogenic tumours, namely CT26 and 4T1. Nevertheless, co-treatment with epigenetic modulating drugs and checkpoint inhibitors improved treatment outcomes, curing more than 80% of the tumour-bearing mice. In this study, animals bearing large CT26 tumours (>600 mm^3^) were treated with anti-PD-1 and anti-CTLA-4 antibodies, in combination with 5-AZA or entinostat. The resulting data showed the eradication of primary tumours in 10 out of 11 mice. Interestingly, the primary tumours and metastases were not found in any of the mice treated with both antibodies plus entinostat, whereas only the primary tumour was detected in the mice treated with both antibodies plus 5-AZA [106]. Preclinical studies suggest that HDAC inhibition induces upregulation of PD-L1, and to a lesser extent PD-L2, in a dose-dependent manner. These results have been obtained in vitro and further confirmed in vivo using a murine B16F10 cell melanoma model. Among the inhibitors evaluated, panobinostat displayed the greatest ability to enhance PD-L1 expression, providing a rationale for panobinostat/anti-PD1 combinatorial treatment [107]. Currently, a better understanding of the molecular mechanisms by which HDACi elicit immunostimulatory effects would contribute to their clinical development as anticancer agents. More recently, in order to test whether HDACi could have a synergistic effect with immunotherapy, panobinostat has been administered in an in vivo B16 melanoma model in combination with T cell transfer therapy [108]. In this study, panobinostat improved the effectiveness of gp100-specific T cell immunotherapy and maintained systemic pro-inflammatory levels. Moreover, it enhanced proliferation, retention, and polyfunctional status of tumour-specific T cells, leading to decreased tumour burden and highly decreased Treg populations. Kroesen et al. have shown that combination of anti-GD2 plus vorinostat reduces NB tumour growth [109]. Further studies have also shown that HDACi upregulate the expression of various components of the immune system, in particular molecules involved in APM as well as those involved in immune co-stimulation. Horing et al. described how TSA, in addition to induction of apoptosis in tumour cells, can augment anti-glioblastoma multiforme (GBM) innate immune responses. Systemic treatment with TSA delayed GBM xenograft growth by enhancing tumour recognition by NK cells [110]. As already aforementioned, HDACi may regulate APM through different ways including activation of class II transactivator (CIITA), a master regulator of MHC II [111]. Additionally, it has been demonstrated that entinostat increased the level of MHC II by transcriptional activation of CIITA in diffuse large B cell lymphoma (DLBCL) [112]. Furthermore, NB and hepatoma cells treated with VPA have shown increased sensitivity to NK cell killing through transcription of MHC class I-related chain A and B (MICA and MICB). In addition, VPA at non-toxic pharmacological concentration arrested growth, induced differentiation, and increased immunogenicity of NB cells through non-toxic mechanisms [113]. Further experiments have been carried out to evaluate the effect of vorinostat on regulation of MICA/B expression. In this study, Yang et al. have reported that vorinostat upregulates the transcription of MICA/B by promoting MICA-associated histone acetylation and by suppressing the MICA/B-targeting miRNAs, such as miR-20a, miR-93, and miR-106b. Vorinostat can regulate miR-17-92 cluster and MCM7 to upregulate MICA expression in hepatoma [114].

Romidepsin displayed an antiproliferative effect on T cells by inhibition of the production of IL-2 and downregulation of CD25 (part of the IL-2 receptor) [115]. Although poor efficacy was observed in the antitumour immune response in vivo, Cao et al. have demonstrated that in vivo treatment with TSA induced suppression of nuclear factor of activated T cells 1 (NFAT1)-regulated FasL expression on activated CD4^+^ T cells. Importantly, they also found that the co-administration of HDACi and anti-CTLA-4 could further enhance the infiltration of CD4^+^ T cells and achieve a synergistic anticancer effect. In fact, within this study, modulation of activation-induced cell death (AICD) of tumour-infiltrating CD4^+^ T cells by TSA enhanced antitumour immune responses, uncovering a novel mechanism underlying the antitumour effect of HDACi [116]. Previous studies have evaluated the capability of rocilinostat (ACY-1215), a HDAC6-specific inhibitor, to prevent skin inflammation through blocking the effector CD8^+^ T cells and impairing the MAPK pathway [117]. Furthermore, since tumour growth induces accumulation of immunosuppressive cells including Tregs, a huge number of studies addressing the effect of HDACi on Tregs and other immunosuppressive cells have been performed. Entinostat inhibited Foxp3 expression and Treg suppressive function in a dose-dependent manner and, at lower doses, enhanced cytokine and vaccine therapies in murine renal cell carcinoma and prostate cancer models, respectively [88]. In contrast, in murine colitis models of inflammation and autoimmunity, the HDAC6-selective inhibitors tubacin and tubastatin A enhanced Treg suppressive function [118]. More studies have revealed that co-treatment with HDACi or DNMTi and checkpoint inhibitors were capable of suppressing MDSCs and eradicate metastatic mouse cancer resistant to immune checkpoint blockade [106]. A very recent study has demonstrated that prostate (LNCAP) and breast (MDA-MB-231) carcinoma cells are more sensitive to T cell-mediated lysis in vitro after clinically relevant exposure to epigenetic therapy with either vorinostat or entinostat and that genetic or pharmacological inhibition studies identified HDAC1 as a key determinant to reverse carcinoma immune escape [119]. Moreover, in two non-epithelial cancers (glioma and mesothelioma), it was found that the epigenetic regulation of the *NY-ESO1* gene requires the sequential recruitment of the HDAC1-mSin3a-NCOR and DNMT1-PCNA-UHRF1-G9a complexes [120].

Treatment with DNMTi allows immunological recognition and cytolysis of cancer cells overcoming the resistance to CTA-specific CTLs. 5-AZA-CdR has been reported to modulate the expression of both CTA and class I human leucocyte antigen (HLA) and the resulting modification in neoplastic cell immunogenicity [121]. 5-AZA has been shown to upregulate PD-L1 in EOC and NSCLC cell lines, eliciting the activation of the viral/IFN response [122]. Nevertheless, another recent report has shown that PD-1 promoter demethylation was associated with PD-1 mRNA upregulation and worse overall response in MDS patients [123]. Notably, patients with MDS resistant to DNMTi showed elevated levels of PD-L1, PD-L2, and CTLA-4, suggesting a putative involvement of PD-1 signalling in resistance mechanisms to hypomethylating agents [124]. Importantly, Odunsi et al. performed a phase I dose escalation of 5-AZA-CdR, in addition to NY-ESO-1 vaccine and doxorubicin liposome chemotherapy, in 12 patients with relapsed EOC. Increased NY-ESO-1 serum antibodies and T cell responses were observed in most patients, encouraging further evaluation in other tumour types [125].

Based on the evidence above highlighted, the cooperation between DNA methylation and histone acetylation in controlling gene transcription prompted some researchers to explore new combined therapies using both HDACi and DNMTi. A synergistic upregulation of MAGE-A genes in selected cancer cell lines by 5-AZA-CdR/TSA combination has been reported [126]. Despite these results, a non-durable synergistic effect was observed for such a combination, with DNMTi remaining the most effective epigenetic drugs in modulating CTA expression in cancer cells. Additional preclinical data confirmed the upregulation of cellular CTA expression by systemic administration of 5-AZA-CdR and modification of class I HLA antigen expression [127]. These in vivo modulations, including NY-ESO expression, were still detectable on melanoma xenografts 30 days after the end of 5-AZA-CdR administration, and injection of BALB/c mice generated high-titre anti-NY-ESO-1 antibodies [127]. Furthermore, 5-AZA-CdR induced demethylation of the Toll-like receptor 4 (TLR4) promoter, an important modulator of the immune response in various cancers, and increased H3K4 trimethylation and Sp1 binding to reactivate silenced TLR4.

In addition, it was demonstrated that the recruitment of the MeCP2/HDAC1 repressor complex increased the low levels of TLR4 expression through epigenetic modification of DNA and histones on the TLR4 promoter in gastric cancer cells [128]. A phase I trial showed that 5-AZA-CdR may be a potential modulator of the immune-activating properties of high-dose IL-2 in melanoma and renal cell carcinoma patients. While upregulation of chemokines and genes involved in IL-1, IL-17, IL-22, and IFN signalling might favour the activity of administered IL-2, downregulation of IL-2Ra, CD3-ε, CD2, and genes involved in IL-2 signalling can be expected to impair IL-2 activity [129].

## Clinical investigation of HDACi or DNMTi treatment in combination with immunotherapies

In the light of the above considerations, researchers have proposed the clinical use of some epigenetic drugs in order to overcome some major limitations of current therapeutic strategies to fight cancer and to evaluate their efficacy and clinical tolerability. Many preclinical studies have been conducted using different classes of HDACi, also corroborated by an increasing number of clinical investigations started by combining HDACi with immunotherapeutics. Immune evasion is the major obstacle to the efficacy of cancer immunotherapies, by preventing long-lasting tumour control. Hence, there is a strong need to restore tumour immune recognition of malignant tumours in order to increase the clinical benefit for patients. HDACi appear to be able to improve the in vivo therapy efficacy, and, although additional preclinical data are needed to assess the efficacy and toxicity of these drugs alone or in combination with other chemotherapeutics and immunotherapy strategies, several clinical studies are being investigated (Table 1). Among current clinical trials, in patients with advanced renal or urothelial cell carcinoma, pembrolizumab and vorinostat will be administered to evaluate the antitumour activity by estimation of serious adverse events (AEs), maximum tolerated dose (MTD), and progression-free survival (PFS). These clinical studies have a run-in phase with sequential single agents and then a combination phase. Thirty patients will be enrolled in two expansion cohorts: 15 anti-PD1-naive patients and 15 anti-PD1-resistant patients (NCT02619253, Table 1) [130]. More interesting evaluation of the potential combined therapy targeting cancer cells will be represented by the study that keeps in consideration the possibility to treat metastatic eye melanoma (PEMDAC) with pembrolizumab and entinostat. Their co-administration will be, respectively, intravenously (IV) for pembrolizumab at 200 mg and orally for entinostat at 5 mg for a period of 24 months. PFS and clinical beneficial rate (CBR) are some of the parameters that should be characterized to evaluate patient responses (NCT02697630, Table 1) [130]. Focusing on DNMTi, 5-AZA or entinostat will be orally administered to metastatic NSCLC patients together with the monoclonal anti-PD1 antibody nivolumab (NCT01928576, Table 1) [130]. In a phase I study, the safety of a combination between orally administered pembrolizumab and 5-AZA will be evaluated (NTC02546986, Table 1) [130]. Likewise, in a phase II study, 60 patients with NSCLC will be enrolled to evaluate the efficacy of 5-AZA-CdR plus nivolumab treatment vs nivolumab alone (NCT02664181, Table 1) [130]. An overview of all other combinations is shown in Table 1.

**Table 1:** Summary of clinical trials describing HDAC and DNMT inhibitors in combination with immune checkpoint blockade therapy [130]

Clinical trial identifier	Status	Phase	Cancer type	Epigenetic drug	Immune checkpoint inhibitor	Additional intervention
NCT02635061	Not yet recruiting	I	Unresectable NSCLC	ACY-241	Nivolumab and ipilimumab	
NCT01686165	Not yet recruiting	II	DLBCL	Belinostat	Rituximab	Yttrium-90
NCT02453620	Recruiting	I	Metastatic unresectable HER2-negative breast cancer	Entinostat	Nivolumab and ipilimumab	
NCT02909452	Recruiting	I	Advanced solid tumours	Entinostat	Pembrolizumab	
NCT03024437	Not yet recruiting	I/II	Advanced cell carcinoma	Entinostat	Atezolizumab	Bevacizumab
NCT02708680	Recruiting	I/II	Breast cancer	Entinostat	Atezolizumab	
NCT02697630	Recruiting	II	Metastatic uveal melanoma	Entinostat	Prembrolizumab	
NCT02805660	Recruiting	I/II	Advanced solid tumours and NSCLC	Mocetinostat	Durvalumab	
NCT02437136	Recruiting	I/II	NSCLC and melanoma	Entinostat	Pembrolizumab	
NCT02993991	Not yet recruiting	I	Squamus cell carcinoma of the oral cavity	Mocetinostat	Durvalumab	
NCT02032810	Recruiting	I	Unresectable stage III/IV melanoma	Panobinostat	Ipilimumab	
NCT01238692	Not yet recruiting	II	DLBCL	Panobinostat	Rituximab	
NCT01282476	Not yet recruiting	II	DLBCL	Panobinostat	Rituximab	
NCT02512172	Recruiting	I	Advanced CRC	Romidepsin and/or 5-AZA	Pembrolizumab	
NCT02538510	Recruiting	I/II	HNSCC and SGC	Vorinostat	Pembrolizumab	
NCT02638090	Recruiting	I/II	Stage IV NSCLC	Vorinostat	Pembrolizumab	
NCT02619253	Recruiting	I/II	Advanced renal or urothelial cell carcinoma	Vorinostat	Pembrolizumab	
NCT02395627	Recruiting	II	Hormone therapy-resistant breast cancer	Vorinostat	Pembrolizumab	Tamoxifen
NCT00667615	Not yet recruiting	I/II	DLBCL	Vorinostat	Rituximab	Cyclophosphamide, etoposide, prednisone
NCT00720876	Not yet recruiting	II	Lymphoma	Vorinostat	Rituximab	
NCT00972478	Not yet recruiting	I/II	DLBCL	Vorinostat	Rituximab	Cyclophosphamide, doxorubicin, prednisone, vincristin
NCT00764517	Not recruiting	II	Lymphoma	Vorinostat	Rituximab	Cladribine
NCT00918723	Not yet recruiting	I	Lymphoma/leukaemia	Vorinostat	Rituximab	Cyclophosphamide, fludarabine
NCT02397720	Recruiting	II	AML	5-AZA	Nivolumab	
NCT02260440	Recruiting	II	Metastatic CRC	5-AZA	Pembrolizumab	
NCT02546986	Recruiting	II	Advanced/metastatic NSCLC	5-AZA	Pembrolizumab	
NCT02508870	Recruiting	I	MDS	5-AZA	Atezolizumab	
NCT02951156	Not yet recruiting	II	DLBCL	5-AZA	Rituximab	Bendamustine, gemcitabine, oxaliplatin
NCT02530463	Recruiting	II	MDS	5-AZA	Nivolumab and/or ipilimumab	
NCT02399917	Recruiting	II	Refractory/relapsed AML	5-AZA	Lirilumab	
NCT02599649	Recruiting	II	MDS	5-AZA	Lirilumab and nivolumab	
NCT02512172	Recruiting	I	MSS advanced CRC	Romidepsin and/or 5-AZA	Pembrolizumab	
NCT02816021	Not yet recruiting	II	Metastatic melanoma	5-AZA	Pembrolizumab	
NCT01928576	Recruiting	I	NSCLC	5-AZA and/or entinostat	Nivolumab	
NCT02795923	Not yet recruiting	II	NSCLC	5-AZA-CdR/tetrahydrouridine	Nivolumab	
NCT02811497	Recruiting	II	Advanced solid tumours	5-AZA	Durvalumab	

Analysing the recent clinical trials, vorinostat and 5-AZA are the drugs most frequently used, likely due to their intense preclinical and clinical investigations.

## Conclusions

Robust data support the role of epigenetic drugs in facilitating immunological targeting of cancer cells by their ability to modulate different mediator factors and pathways involved in the interaction between tumour cells and the immune system. Following this observation, HDACi or DNMTi have been combined with immune checkpoint therapies to provide more significant benefit for cancer patients than monotherapy. In this review, we have summarized preclinical and clinical results combining HDACi or DNMTi with immune checkpoint inhibitors and their direct effects on various components of the immune system. Although more and more preclinical trials are being conducted to enhance safety and efficacy, especially for DNMTi, these findings will help along the road for the discovery and the development of novel therapeutic approaches in cancer immunotherapy. Focused on the results from preclinical studies of HDACi on Tregs, either class I or class II HDAC inhibition may have opposite effects on Treg function as inhibition or promotion, respectively. Thus, it is currently believed that cancer treatments with class I-specific HDACi could provide future interesting outcomes in patients. Indeed, different studies have shown that immunomodulatory effects of HDAC inhibition with high specificity may lead to a selective immune regulation, when compared with pan-HDACi treatment. Even if selective HDACi may provide greater efficacy, the identification of the proper dose could reduce the adverse effects associated with HDAC inhibition. Regardless of which epigenetic modulator is used in preclinical or clinical studies, the toxicity on different tumours types remains a great challenge. A better understanding of the molecular mechanisms by which HDACi and DNMTi elicit immunomodulatory effects could help to ameliorate their clinical development. Nevertheless, to achieve beneficial responses in patients, a deep investigation on the main molecular processes on which the immune system relies remains of high interest. Future inquiry on immuneregulatory mechanisms could provide more interesting targets for epigenetic drugs in order to improve cancer cell recognition by T cells and overcome cancer therapy failure. In addition, it could be interesting to evaluate the immunomodulatory activity of other epigenetic modulators (i.e. HMTs and demethylase inhibitors), even highlighting their clinical effects by combination with the already described HDACi or DNMTi.

## Abbreviations

5-AZA: Azacitydine
5-AZA-CdR: Decitabine
AEs: Adverse events
AICD: Activation-induced cell death
AML: Acute myeloid leukaemia
APC: Antigen-presenting cell
APL: Acute promyelocytic leukaemia
APM: Antigen presentation machinery
BATF: Basic leucine zipper transcription factor
cAMP: Cyclic adenosyl monophosphate
CBR: Clinical beneficial rate
CD28: Cluster of differentiation 28
CIITA: Class II transactivator
CMML: Chronic myelomonocytic leukaemia
CTA: Cancer testis antigen
CTCL: Cutaneous T cell lymphoma
CTL: Cytotoxic T lymphocytes
CTLA-4: Cytotoxic T lymphocyte-associated antigen-4
DC: Dendritic cell
DLBCL: Diffuse large B cell lymphoma
DNMT: DNA methyltransferase
DZNeP: Deazaneplanocin A
EGFR: Epidermal growth factor receptor
EMA: European Medicines Agency
EOC: Epithelial ovarian cancer
EZH2: Enhancer of zeste homolog 2
FOXO1: Forkhead box protein O1
Foxp3: Forkhead box protein 3
GC: Gastric carcinoma
HATs: Histone acetyltransferases
HDAC: Histone deacetylase
HLA: Human leucocyte antigen
HMTs: Histone methyltransferases
IFN-γ: Interferon-γ
IL-6: Interleukin-6
iTreg: Induced regulatory T
lncRNAs: Long noncoding RNAs
MAbs: Monoclonal antibodies
MAGE-A: Melanoma-associated antigen
MAPK: Mitogen-activated protein kinases
MCL: Mantle cell lymphoma
MDS: Myelodysplastic syndromes
MDSCs: Myeloid-derived suppressor cells
MHC: Major histocompatibility complex
MICA: MHC class I-related chain A
MICB: MHC class I-related chain B
miRNAs: MicroRNAs
MITF: Microphthalmia-associated transcription factor
MM: Multiple myeloma
MTD: Maximum tolerated dose
NB: Neuroblastoma
NEAT1: Nuclear paraspeckle assembly transcript 1
NFAT1: Nuclear factor of activated T cells 1
NF-κB: Nuclear factor kappa light chain enhancer of activated B cells
NK: Natural killer
NSCLC: Non-small cell lung cancer
PD: Programmed cell death protein
PFS: Progression-free survival
PRAME: Preferentially expressed antigen in melanoma
PRC2: Polycomb repressive complex 2
PTCL: Peripheral T cell lymphoma
Rb1: Retinoblastoma 1
RORγT: RAR-related orphan receptor
ROS: Reactive oxygen species
Sin3A: Transcription regulator family member A
Sp1: Specificity protein 1
STAT4: Signal transducer and activator of transcription 4
TAA: Tumour-associated antigen
TAM: Tumour-associated macrophage
Tbx21: T-box transcription factor 21
TCR: T cell receptor
TGF-β: Transforming growth factor β
Th: T helper
Tim-3: T cell immunoglobulin and mucin domain 3
TKIs: Tyrosine kinase inhibitors
TLR4: Toll-like receptor 4
TME: Tumour microenvironment
TNF: Tumour necrosis factor
Treg: T regulatory cell
TSA: Trichostatin A
VDAC1: Voltage-dependent anion- selective channel protein 1
VEGF: Vascular endothelial growth factor
VPA: Valproic acid
